# Resonant signals in the lithosphere–atmosphere–ionosphere coupling

**DOI:** 10.1038/s41598-022-18887-1

**Published:** 2022-08-26

**Authors:** Chieh-Hung Chen, Yang-Yi Sun, Xuemin Zhang, Yongxin Gao, Fei Wang, Kai Lin, Chi‑Chia Tang, Rong Huang, Rui Xu, Jing Liu, Yali Wang, Cong Chen

**Affiliations:** 1 Institute of Geophysics and Geomatics, China University of Geosciences, Wuhan, 430074 China; 2 Institute of Earthquake Forecasting, China Earthquake Administration, Beijing, 100036 China; 3 Applied Institute of Mechanics, School of Civil Engineering, Hefei University of Technology, Hefei, 230009 China; 4 Department of Geological Engineering, Chengdu University of Technology, Chengdu, 610059 China; 5 Sichuan Earthquake Bureau, Chengdu, 610041 China; 6 China Earthquake Networks Center, Beijing, 100045 China

**Keywords:** Geophysics, Solid Earth sciences, Space physics

## Abstract

A study in the lithosphere, atmosphere and ionosphere (LAI) coupling often troubles scientists due to a certain distance between distinct instruments, which monitor geophysical parameters in different spheres. An instrumental system was established in southwest China (Leshan; LESH) for monitoring vibrations and perturbations in LAI (MVP-LAI). A ground-based Global Navigation Satellite System (GNSS) receiver at the YADU station locates ~ 260 km away that continuously receives electromagnetic signals transmitted from the BeiDou navigation System (BDS) geostationary satellites to monitor the total electron content (TEC) at the ionospheric pierce point right over the MVP-LAI system. The employment of YADU TEC benefits in elimination of possible shaking effects happening on multiple instruments at the LESH station and mitigation the troubles due to the discrepancy in observation places. Through a stacking process on the retrieved data for increase of signal to noise ratios, a novel phenomenon of the resonant LAI coupling at a fundamental mode of ~ 3.4 mHz and its multiples persists in ground vibrations, atmospheric pressure and TEC retrieved from the MVP-LAI system and the YADU station. The retrieved data share frequencies during the operational period of 1.5 months that is irrelevant to obvious events in the lithosphere, atmosphere and ionosphere. The persistence of the resonant LAI coupling is essential in the Earth’s system.

## Introduction

Interactions in the lithosphere, atmosphere and ionosphere (LAI) are important to creatures living on the Earth. Variations in one sphere of LAI can dominate changes in the other two spheres. Phenomena of the LAI coupling generated by distinct types of events (e.g., pre-earthquake anomalies^1–4^, co-seismic responses^5–10^, tsunami^11,12^, volcano eruptions^13–15^ and so on^16,17^) near the Earth’s surface have been widely studied in recent decades. The LAI coupling before earthquakes is wishful from seismo-TEC (total electron content) anomalies in the ionosphere without obvious vibrations near the Earth’s surface. Hayakawa^18,19^ reported that seismo-TEC anomalies could originate from seismo-chemical, -electrical conductivity, -thermal, and -electromagnetic anomalies close to epicenters. In contrast, the coupling is often attributed to pronounced uplift and depression of the Earth’s surface and/or sea water levels due to atmospheric resonance and/or acoustic waves^10,11,14,20^. Previous studies suggested that Rayleigh waves and volcanic eruptions at frequencies of ~ 4 mHz induce atmospheric resonance that contributes to the coupling^7,14,21^. The frequencies with pronounced amplitudes retrieved from ground vibrations overlap the fundamental mode of ionospheric TEC^14^. On the other hand, acoustic waves in the atmosphere can originate from Rayleigh waves and tsunami^10,11^. Acoustic waves take ~ 10 min upward propagating to the ionosphere driving changes in TEC at an altitude of ~ 350 km^10,11^. The atmospheric gravity wave (AGW) is another major factor that contributes to the coupling^22,23^. Those results are typically studied by utilizing TEC data in the ionosphere retrieved from ground-based Global Navigation Satellite System (GNSS) receivers and seismic data recorded by seismometers on the Earth’s surface. In general, seismometers record ground vibrations with high temporal resolutions at a certain location. However, the TECs are generally derived by utilizing signals transmitted from orbiting satellites. The derived TECs are not above a particular location but along ground tracks of the ionopsheric pierce points (IPPs) travelling along the time^24^. A horizontal distance often exists between the ground tracks of TECs and the particular location of seismometers. Influence resulted from the discrepancy in observation places (i.e., travelling ground tracks and the particular location) for the distinct geophysical parameters (i.e., the TECs and ground vibrations) cannot be efficiently mitigated and is generally ignored.

**Figure 1:**
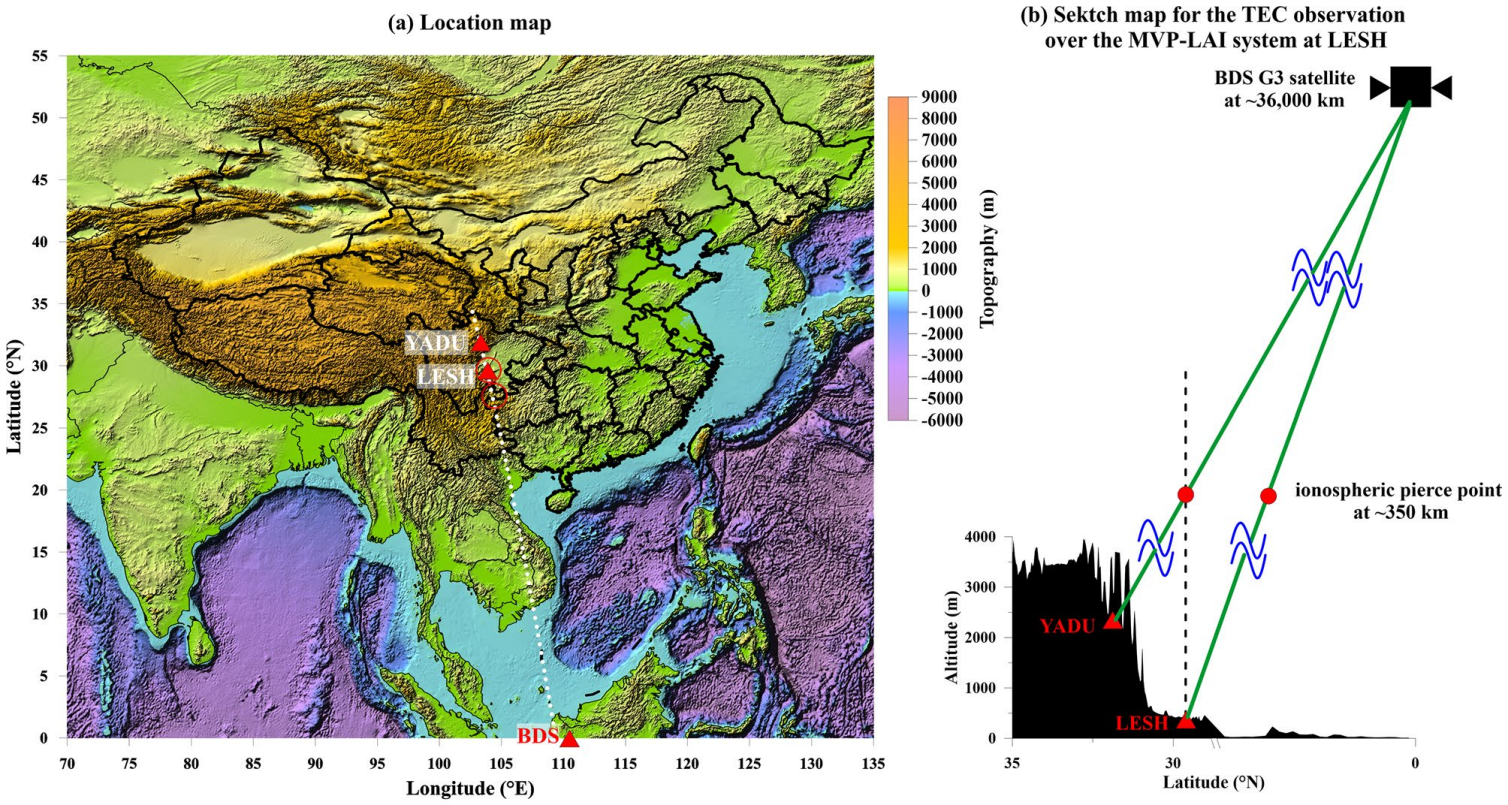
Locations of the LESH (MVP-LAI) and YADU stations and the sketch map for the TEC observation. The location map for the stations lying on the topography and the sketch map for the TEC observation are shown in (**a,b**), respectively. The MVP-LAI instrumental system is located at LESH. The employment of the BDS G3 satellite is located at (0°N, 110.5°E). The other ground-based GNSS receiver is located at YADU for fixing the ionosphere pierce point above the MVP-LAI system. The red open circles indicate the locations of IPPs for the LESH and YADU stations. The profile along the white dots crossing YADU, LESH and the BDS GEO (denoted by the red triangles) in (**a**) is shown in (**b**). The green lines show that electromagnetic signals from the BDS G3 satellite received by two ground-based receivers at the LESH and YADU stations. The black dashed line indicates a direction vertically injected from the LESH station to the ionosphere. An intersection between the green line and black dashed line is the ionospheric pierce point with an altitude of ~ 350 km right above the MVP-LAI system.

A novel instrumental system has been established in Leshan (LESH, 29.6°N, 103.9°E), Sichuan, China^25^. The system was designed to Monitor Vibrations and Perturbations in the LAI (MVP-LAI) and officially operated since September 2021 (Fig. 1a). The MVP-LAI system locates near the Qinghai-Tibet plateau. A pronounced discrepancy of ~ 3000 m in altitude between the system and the plateau creates an excellent natural environment for studying the LAI coupling (Fig. 1b). Achievements of the MVP-LAI system are not limited within a specific topic but widely face numerous scientific issues associated with the coupling. The system comprises 14 district instruments in the current stage. Most instruments were installed within a place of ~ 400 m^2^ for monitoring more than 20 distinct geophysical parameters with bare influence caused by the discrepancy in observation places. In the MVP-LAI system, the TECs are retrieved from electromagnetic signals at dual frequencies transmitted from the geostationary (GEO) satellites operated by the BeiDou navigation System (BDS)^26^. The BDS GEO satellites are hanged at altitude of ~ 36,000 km above the Earth. The employment of the GEO satellites leads the derived TECs at a particular place over the Earth’s surface 24 h a day due to almost motionless of the IPPs and high elevation angles. Kunitsyn et al.^27^ reported that the BDS GEO satellites provide a relatively-low noise level for the TEC researches. We thus designed a novel experiment by utilizing the BDS GEO satellites together with multiple instruments in the MVP-LAI system.

In this study, we collected ground vibrations from broadband seismometers, atmospheric pressure from barometers, and TECs from the ground-based GNSS receivers at the MVP-LAI system and the YADU station (31.9°N, 103.4°E) for examinations of the LAI coupling during 1 August 2021–15 September 2021 (Fig. 1a). Note that electromagnetic signals, which are transmitted from the BDS G3 satellite (0°N, 110.5°E), received by the MVP-LAI system and the YADU station are utilized for the examinations. The IPP at the altitude of 350 km for the ground-based GNSS receiver at the MVP-LAI system is located southeast ~ 250 km away from the system (Fig. 1). In contrast, the IPP for the receiver at the YADU station is located right over the MVP-LAI system. These retrieved data (i.e., ground vibrations, air pressure, and TECs) were separated into numerous segments in the temporal domain. Each segment was transferred into the frequency domain via the Fourier transform for the following process of superimposition. The superimposition benefits in mitigating the influence from the sudden events for retrievals of resonant signals. Resonance characteristics for distinct data are investigated, and are further utilized to examine the phenomena of the LAI coupling during the study period.

## Methodology

Instead of traditional methods for investigation of amplitude enhancements in the frequency domain, we retrieved resonant signals from a simple process for adaption of resonance nature. The analytical data (i.e., ground vibrations, atmospheric pressure, and TECs) were obtained from the LESH and YADU stations. These time series data were separated into numerous segments by utilizing a moving window of 5000 s with a short step of 2% dataset in the time domain. In other word, daily data are separated into 815 segments. Separated data in each segment are transferred into the frequency domain by utilizing the Fourier transform. We determine amplitude increases and decreases at each frequency for each segment. Hereafter, we count the total numbers for the increases and the decreases at each frequency from all the transferred segments in one day, based on an assumption of the equal contribution between the increase and decrease regardless quantification of the amplitudes. The occurrence indicator (OI) is defined as a difference between the total counts for the increase and the decrease at each frequency. The OI with a significant positive value suggests that a frequency characteristic often exists in analyzed data. In contrast, a negative OI value or OI near zero suggest that the characteristic is obscure.

### Analytical results

**Figure 2:**
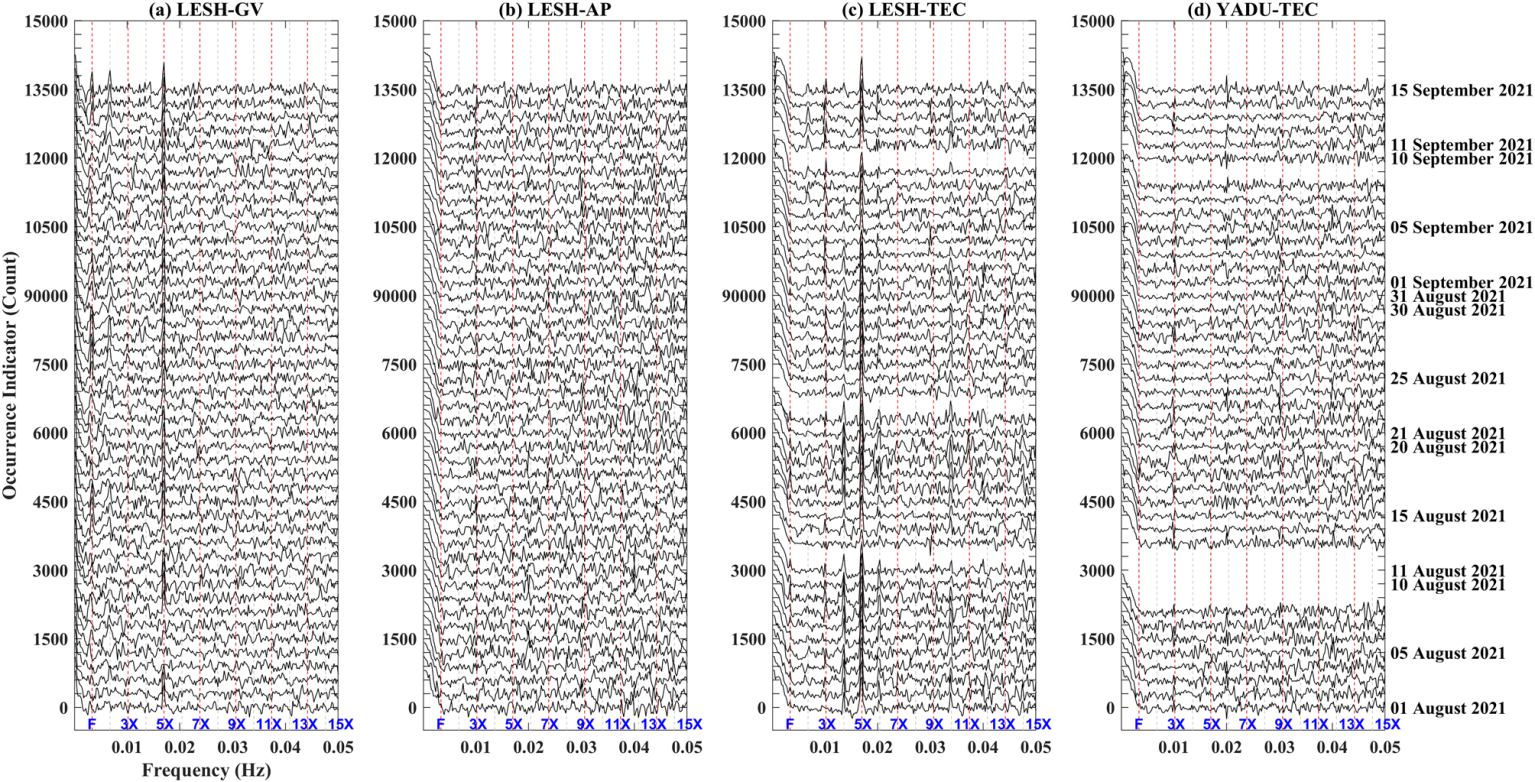
The occurrence indicators function as frequencies for the ground vibrations, atmospheric pressure and TEC at the LESH and YADU station from 1 August 2021 to 15 September 2021. The ground vibrations (GV), atmospheric pressure (AP) and TEC at the LESH station are shown in (**a–c**), respectively. The TEC at the YADU station is shown in (**d**). The vertical dashed lines indicate the fundamental frequency and its multiple ones. The blue mark of “F” denotes the fundamental frequency of 3.4 mHz. The other marks of “X” following a number denote the multiple frequencies to the fundamental mode. Note that occurrence indicators increase with a step of 500 for days after 1 August 2021.

The OI values for ground vibrations, atmospheric pressure, and TECs at the MVP-LAI system from 1 August 2021 to 15 September 2021 are shown in Fig. 2. Ground vibrations have large OI values at frequency of ~ 3.4 mHz and its multiples at ~ 6.8 mHz, and ~ 17 mHz (Fig. 2a). The OI values for the triple and quadruple frequencies of ~ 3.4 mHz are smaller than the others (i.e., ~ 3.4 mHz, ~ 6.8 mHz, and ~ 17 mHz) due to unknown factors. Regarding with the pressure, the OI values are insignificant at frequency of ~ 3.4 mHz (Fig. 2b). In contrast, significant OIs concentrate at frequency of ~ 10.2 mHz that is triple of 3.4 mHz. In terms of the TEC data from the MVP-LAI system, pronounced OIs often distribute at frequency of ~ 17 mHz (Fig. 2c). Meanwhile, pronounced OIs can be usually found at frequencies of ~ 10.2 mHz, ~ 13.6 mHz, ~ 20.4 mHz, and higher. Notably, these frequencies are also the multiple frequencies of 3.4 mHz. These data share frequencies that are suspected to be caused by shaking effects on barometers and ground-based GNSS receivers due to ground vibrations. For further examining the shaking effects, the TEC data recorded by the YADU station are taken into for comparison. The YADU station locates ~ 200 km away from the MVP-LAI system. The IPP of the TEC is above the MVP-LAI system. The YADU TEC exhibits pronounced OIs at frequencies of ~ 10.2 mHz and ~ 20.4 mHz (Fig. 2d). The YADU OIs are weak at frequencies of ~ 17 mHz and ~ 13.6 mHz due to unknown reasons. The pronounced OI values of TEC at two frequencies of ~ 10.2 mHz and ~ 20.4 mHz between the MVP-LAI system and YADU stations are comparable. The comparable results of the two distant stations suggest that the shaking effects are irrelevant factors dominating the obvious OIs. In short, the frequencies (i.e., ~ 3.4 mHz and its multiple frequencies) are nature and exist in our recorded distinct geophysical parameters.

## Discussions and conclusion

The MVP-LAI system together with the YADU station formed a novel geometry that observed the phenomenon of the LAI resonant coupling. If the resonance is nature, the dominant source can exist in any sphere. Additional examinations were processed for further investigating potential factors, including artificial, solar, ionospheric, satellite, lithospheric effects, associated with the LAI resonant coupling. It is well known that artificial harmonics can be caused by the Fourier transform. We thus analyzed the TEC data utilizing the Hilbert-Huang transform (HHT) to eliminate the artificial harmonics of the Fourier transform effect (Fig. S1). The enhancements of the TECs can be consistently observed at the frequency of ~ 15 mHz (Fig. S1) that roughly agrees with the large OI values of ~ 13.6 mHz from the Fourier transform (Fig. 2c). This suggests that the resonant signals do not caused by the Fourier transform (artificial) effect.

Magnetic storms are one kind of the space weather events that often excite auroras in the polar zones. Once the coupling is caused by the magnetic storm, the duration of the coupling should equal to and be limited within the duration of the storm. The duration of the coupling observed in this study is ~ 45 days. The phenomenon persists ~ 45 days that is not dominated by magnetic storms (the solar effect). Similarly, no well-known ionospheric event persists ~ 45 days. Therefore, the factors of the solar and ionospheric effects can be excluded. On the other hand, Tape et al.^28^ reported that the auroral events trigger variations in geomagnetic data and seismograms at the long period (40–800 s). Auroras are not the major factors triggering the coupling observed in this study due to that the YADU and LESH stations are far away from the polar zones.

Furthermore, we retrieved the TEC data from the other four ground-based GNSS receivers located > 1000 km away from the MVP-LAI system for examining whether the resonant phenomenon relates to the satellite effect (Fig. S2). The locations of the IPPs for the four receivers are distributed over the particular places near (38°N, 102.5°E) and (22.5°N, 105°E) shown in Fig. S2. Both the particular places align the white-dot line crossing YADU, LESH, and the BDS G3 satellite (Fig. 1). The TEC data from the same satellite (i.e., BDS G3) at the IPPs exhibit resonant characteristics at ~ 10.2 mHz, ~ 20.4 mHz, and ~ 30.6 mHz over the southern place (i.e., 22.5°N, 105°E) and no pronounced signature over the northern place (i.e., 38°N, 102.5°E) (Fig. S3). This suggests that the resonant phenomenon is not dominated by the BDS G3 satellite. To double check that the BDS G3 satellite is not the major factor of the resonant phenomenon, the TEC data from the BDS G2 satellite at the IPPs over the particular places are retrieved from distinct ground-based receivers for further examinations (Fig. S3). Similarly, the pronounced resonant phenomenon is observed limited in the south place. This verifies that the resonant phenomenon is less relevant to the satellite effect. Note that we also computed the TECs at the LESH and YADU stations utilizing the signals transmitted from the BDS G5 satellite (Fig. S4). The OI index exhibits characteristics without obvious resonant signals from the BDS G5 satellite (Fig. S4). The results suggest that the resonant phenomenon exists within a limited area in the Earth’s system, and is consistent with the observation from the ground-based GNSS receivers being located a certain distance (> 1000 km) away from the MVP-LAI system (Figs. S2 and S3).

Based on the examination above, we suspect that the resonant coupling would be contributed by the lithospheric effect. In general, the LAI coupling is mainly triggered by intense temporary vibrations near the Earth’s surface. The temporary vibrations can be triggered by Rayleigh waves, tsunami, and volcano eruptions^7,10,11,14,21^. However, in this study, the frequencies with the obvious OIs are lower than the frequencies of intense ground vibrations due to earthquakes, and are higher than the frequencies associated with atmospheric gravity waves (AGWs), traveling ionospheric disturbances (TIDs), and even tides e.g.^10,29,30^. The LAI coupling without significant ground vibrations observed in this study inspires us to think about possible physical mechanisms behind. Previous studies^7,14,21^ reported that ground vibrations with characteristics of frequency at ~ 4 mHz can induce atmospheric resonance that contributes to the coupling. However, instead of observations of resonant signals, enhancements of amplitude at ~ 4 mHz are directly referred the resonance. No matter the enhancements and/or the resonance, the frequency at ~ 4 mHz is the essential of the resonant LAI coupling. The fundamental mode at the frequency of ~ 3.4 mHz observed in Fig. 2 is comparable with the reports of the atmospheric resonance in the previous study. Therefore, the resonance of ground vibrations can contribute the LAI coupling. The other problem is how the coupling originates from insignificant ground vibrations. Chou et al.^31^ reported that the TEC perturbations can be generated from the typhoon that persistently perturbs the atmosphere. Figure 2a shows that the resonant signals persist in ground vibrations at last 1.5 months. We suppose that the persistent ground vibrations are capable of modulating variations in atmosphere and sequentially changing TEC in the ionosphere. A M6 earthquake occurred around the MVP-LAI system on 16 September 2021^32^. Pre-earthquake ground vibrations and crustal deformation persist a few months in a wide area that has been reported^33–36﻿^. The forthcoming earthquake would be a promising source providing persistent ground vibrations in a wide area and causing the resonant coupling shown in this study.

In short, we exclude disturbances from lateral areas by installing instruments within a limited place. The TEC data retrieved from the ground-based GNSS receiver at the YADU station, which is ~ 200 km away from the instrumental array, are utilized for eliminating influences of possible shaking effects on these instruments. Characteristics of the resonance at the particular frequencies can be persistently found from distinct geophysical parameters during the operational (1.5-month) period of the MVP-LAI system. The observation results suggest that the resonant signals are essential in the Earth’s system coupling in LAI. The resonant frequencies for ground vibrations in the lithosphere overlap the fundamental mode (~ 3.4 mHz) of the pressure in the atmosphere and TEC in the ionosphere. The resonant coupling can be attributed to persistent ground vibrations that gradually modulate the atmospheric pressure and the ionospheric TEC. Causal mechanisms of resonant ground vibrations are not fully understood. Investigation of the resonant LAI coupling sheds lights on studying interaction in the Earth’s system.

## Supplementary Information

Supplementary Information.

## Data Availability

Data are available at the link https://doi.org/10.6084/m9.figshare.18166574.v1.
