# Supplementary material for: Resonant signals in the lithosphere–atmosphere–ionosphere coupling
Source: Sci Rep. 2022 Aug 26;12:14587. doi: 10.1038/s41598-022-18887-1 (PMC9418318; doi:10.1038/s41598-022-18887-1)
Supplement: Supplementary file 1 — Supplementary Information. [file 41598_2022_18887_MOESM1_ESM.docx]

Supporting Information for

**Resonant signals in the lithosphere-atmosphere-ionosphere coupling**

Chieh-Hung Chen^1,*^, Yang-Yi Sun^1^, Xuemin Zhang^2^, Yongxin Gao^3^, Fei Wang^4^, Kai Lin^1^, Chi‑Chia Tang^1^, Rong Huang^1^, Rui Xu^5^, Jing Liu^2^, Yali Wang^6^, Cong Chen^5^

^1^Institute of Geophysics and Geomatics, China University of Geosciences, Wuhan 430074, China, ^2^Institute of Earthquake Forecasting, China Earthquake Administration, Beijing 100036 China, ^3^Applied Institute of Mechanics, School of Civil Engineering, Hefei University of Technology, Hefei, 230009, China, ^4^Chengdu University of Technology, Chengdu 610059, China, ^5^Sichuan Earthquake Bureau, Chengdu 610041, China, ^6^China Earthquake Networks Center, Beijing 100045, China

**Contents of this file**

The file contains three figures (Figures S1–S4).

**Introduction**

Figure S1 shows the amplitude of the TECs as a function of frequencies via the Hilbert-Huang transform for eliminating the Fourier transform effect. Figure S2 shows location map of the ground-based GNSS receivers and associated IPPs in the supplementary. Furthermore, we retrieved the TEC data at the two selected IPPs over the particular places near (38°N, 102.5°E) and (22.5°N, 105°E) for the BDS G2 and BDS G3 satellites to support the resonant phenomenon observed at the YADU station (Figure S3). We also demonstrate the occurrence indicators associated with the TECs at the LESH and YADU stations for the BDS G5 satellite to examine whether the resonant phenomena are dominated by the particular satellite or not (Figure S4).


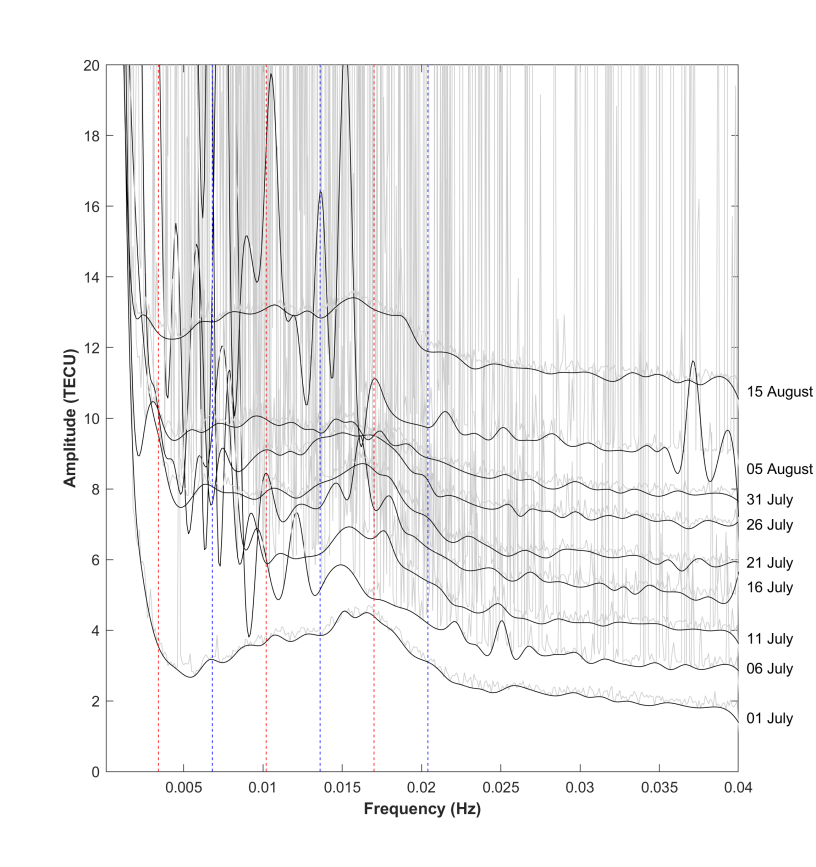


Figure S1. The amplitudes of the TECs as a function of frequencies computed from the Hilbert-Huang transform. The grey lines show the amplitudes of the TECs as a function of frequencies on a particular date marked at the end of the lines. The black lines are the lower envelops of the grey lines. Vertical red and blue dashed lines denote the fundamental frequency of 3.4 mHz and its multiple ones.


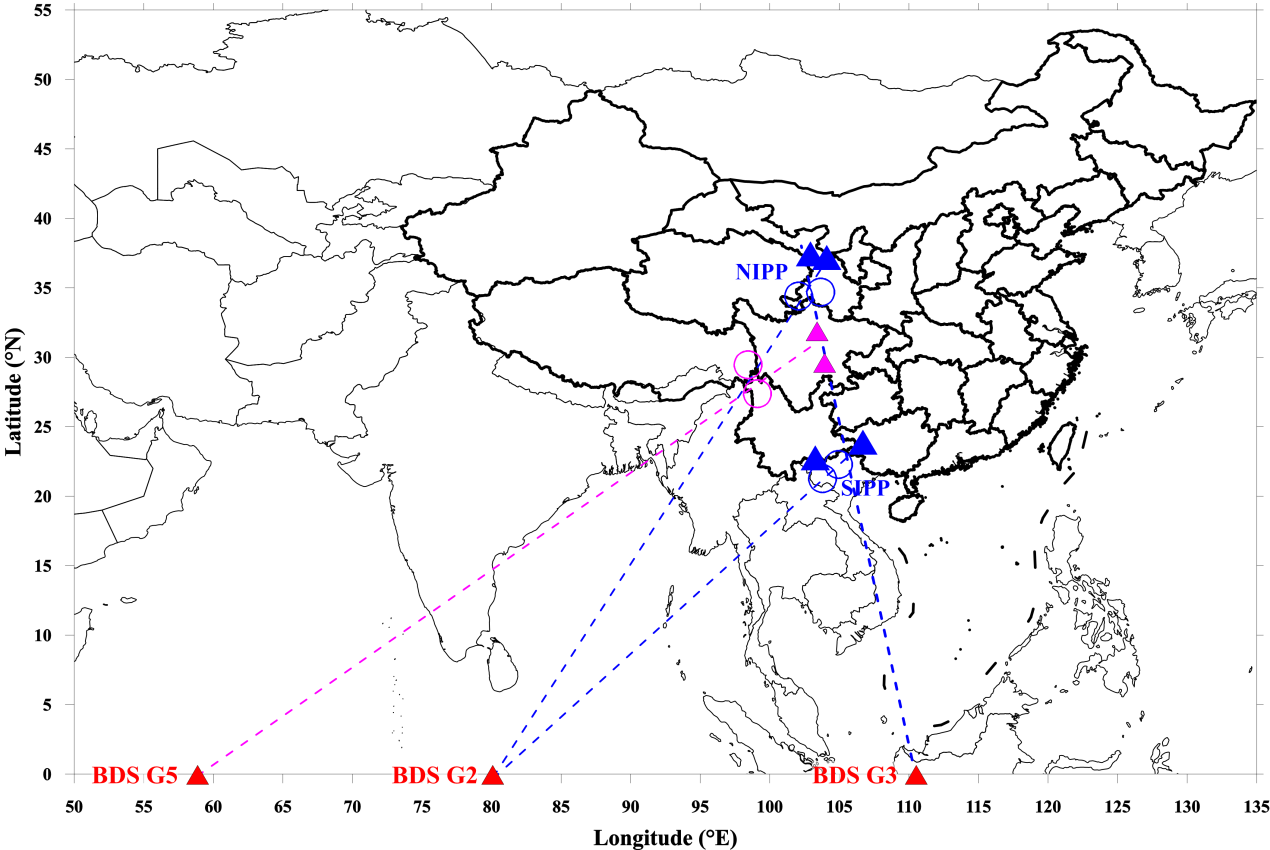


Figure S2. The location map of the ground-based GNSS receivers and associated IPPs in the supplementary. The triangles denote the locations of the ground-based GNSS receivers and the GEO BDS satellites. The open circles show the locations of the IPPs. The blue and pink colors show the receivers and IPPs for Figures S3 and S4, respectively. The NIPP and SIPP are the northern and southern places for the IPPs examined in this study. The dashed lines indicate a set of the ground-based receivers, the IPPs and the satellites.


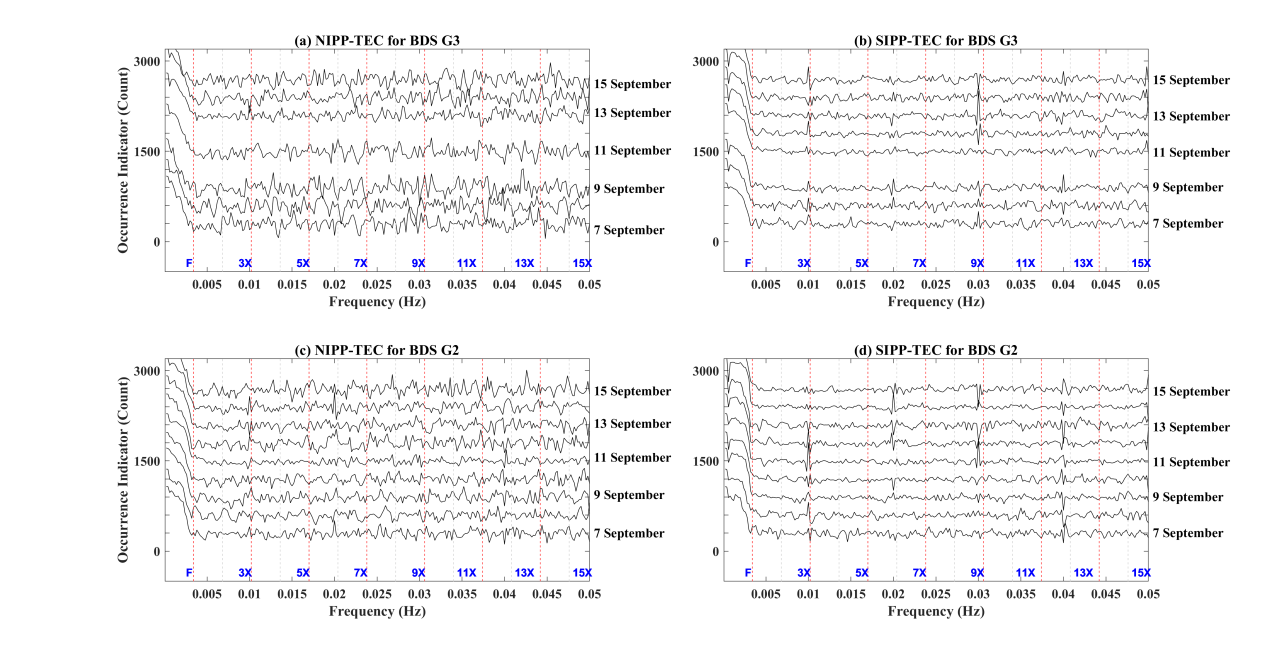


Figure S3. The occurrence indicators function as frequencies for the TEC data at the two selected IPPs from the BDS G3 and BDS G2 satellites during 7 September 2021–15 September 2021. Northern and southern IPPs (i.e., NIPP and SIPP) are located near (38°N, 102.5°E) and (22.5°N, 105°E), respectively. The TECs at the two selected IPPs for the BDS G3 satellite are shown in (a) and (b). In contrast, the TECs at the two selected IPPs for the BDS G2 satellite are shown in (c) and (d). The vertical dashed lines indicate the fundamental frequency and its multiple ones. The blue mark of “F” denotes the fundamental frequency of 3.4 mHz. The other marks of “X” following a number denote the multiple frequencies to the fundamental mode. Note that occurrence indicators increase with a step of 300 for days after 1 August 2021.


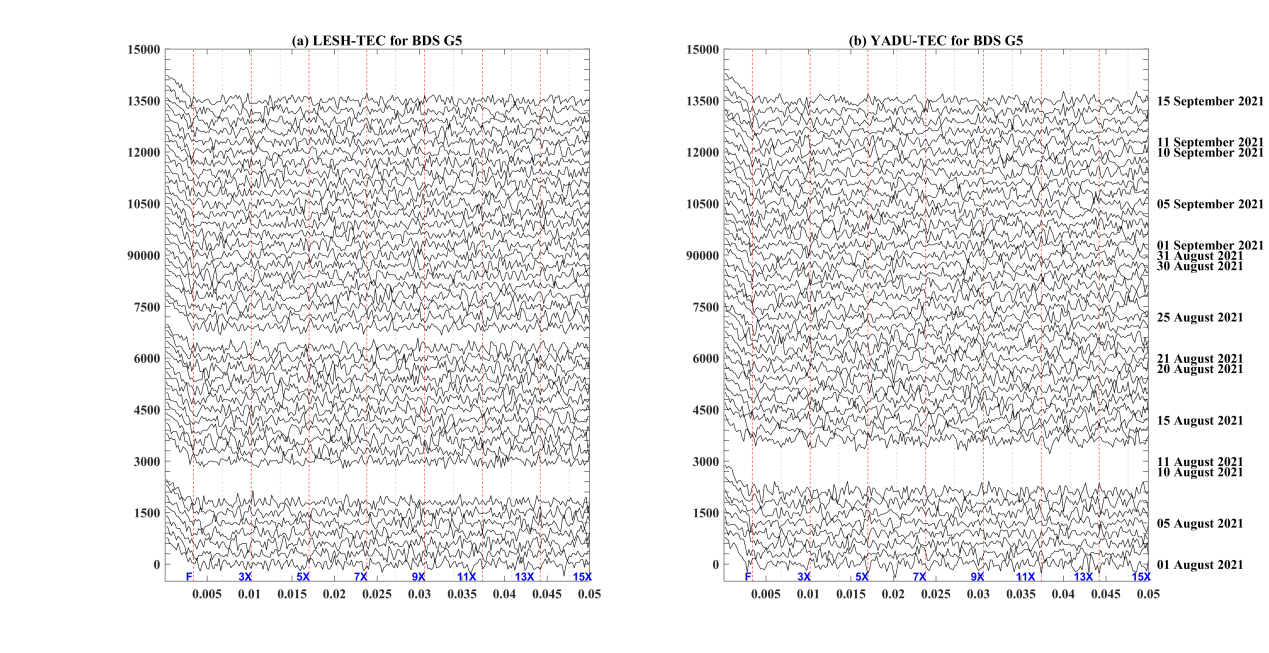


Figure S4. The occurrence indicators function as frequencies for the TEC data at the LESH and YADU station from the BDS G5 satellites during 1 August 2021–15 September 2021. The TEC at the LESH and YADU station are shown in (a) and (b), respectively. The vertical dashed lines indicate the fundamental frequency and its multiple ones. The blue mark of “F” denotes the fundamental frequency of 3.4 mHz. The other marks of “X” following a number denote the multiple frequencies to the fundamental mode. Note that occurrence indicators increase with a step of 500 for days after 1 August 2021.
